# Formulation and Evaluation of Astaxanthin-Loaded Invasomes as Therapeutic Approaches for Alzheimer’s Disease Induced in Rats: Role of SIRT-1/BDNF/miRNA-134/GSK-3β Signaling

**DOI:** 10.1007/s12035-025-05241-5

**Published:** 2025-08-01

**Authors:** Mohamed A. Kandeil, Eman T. Mohammed, Marwa A. Ibrahim, Rania A. Radi, Amr Gamal, Abdel-Razik H. Abdel-Razik, Fatma Khalil, Dina Sabry

**Affiliations:** 1 Department of Biochemistry, Faculty of Veterinary Medicine, Beni-Suef University, Beni-Suef, 62511 Egypt; 2 Department of Biochemistry and Molecular Biology, Faculty of Veterinary Medicine, Cairo University, Giza, 12211 Egypt; 3 Department of Pharmaceutics and Industrial Pharmacy, Faculty of Pharmacy, Beni-Suef University, Beni‑Suef, Egypt; 4 Department of Histopathology, Faculty of Veterinary Medicine, Beni-Suef University, Beni-Suef, 62511 Egypt; 5 Animal and Poultry Management and Wealth Development Department, Faculty of Veterinary Medicine, Beni-Suef University, Beni-Suef, 62511 Egypt; 6 Department of Medical Biochemistry and Molecular Biology, Faculty of Medicine, Badr University in Cairo, Cairo, 11829 Egypt; 7 Department of Medical Biochemistry and Molecular Biology, Faculty of Medicine, Cairo University, Cairo, 11562 Egypt

**Keywords:** Alzheimer’s disease, Astaxanthin invasome, MiRNA-134, SIRT-1, Neurotoxicity

## Abstract

Alzheimer’s disease (AD) is a progressive age-dependent neurodegenerative disorder associated with oxidative brain damage, disrupted neuronal transmission, memory loss, and behavioral changes, with aluminum being a key environmental risk factor that exacerbates its effect. The aim of this study is to enhance the therapeutic potential of astaxanthin (AST) in Alzheimer’s disease by formulating it into invasomal carriers, with special emphasis on SIRT-1/BDNF/miRNA-134/GSK-3β signaling in an AD-like rat model caused by aluminum chloride (AlCl_3_) at a dose of 100 mg/kg/day for 60 days. Optimum AST-loaded invasomes (AST-LI) were prepared using a formulation of phospholipid: ethanol: cineole as 300 mg: 0.3 ml: 0.1 ml for the production of stable vesicles with high entrapment efficiency and negative zeta potential indicating good stability and de-aggregation. As a SIRT-1 activator, AST-LI supplementation improved learning and memory by alleviating the brain redox status (reduced glutathione; GSH, malondialdehyde; MDA), mitochondrial dysfunction, and inflammatory response linked to amyloid β (Aβ) clearance and GSK-3β-mediated p-tau inhibition. It enhanced both spatial and non-spatial short-term memory in rats and restored neurotransmitter levels by raising serotonin and reducing acetylcholinesterase (AChE) and monoamine oxidase (MAO) activities in the brain. Furthermore, AST-LI significantly restored the brain recovery proteins such as Chemokine C-X3-C motif ligand 1 (CX3CL1), glial fibrillary acidic protein (GFAP), brain-derived neurotrophic factor (BDNF), and miRNA-134. These modulations may underlie the observed improvements in oxidative stress, inflammation, apoptosis, and histological outcomes in the AD-like model. In conclusion, improved AST-LI formulations represent promising therapeutic approaches for AD by modulating SIRT-1/BDNF/miRNA-134/GSK-3β signaling.

## Introduction

Alzheimer’s disease (AD) is a chronic neurodegenerative illness and a leading cause of dementia, marked by selective death of neurons and impairment of cognition brought on by the buildup of amyloid β (Aβ) protein plaque and hyperphosphorylated tau proteins [1]. Its prevalence is increasing, with an estimated 44 million cases reported in 2015 and predictions that it will double by 2050, making it a severe worldwide health concern [2]. Genetic and environmental factors contribute to its development; however, the exact etiology is still unknown. Aluminum (Al), a well-known industrial and environmental neurotoxin, is linked to the pathophysiology of AD [3] by altering the structure of neurons, interfering with neurotransmission and blood–brain barrier (BBB) permeability, and causing inflammation that results in aberrant synapses and memory loss [4]. It has been demonstrated that exposure to aluminum chloride (AlCl_3_) speeds up Aβ aggregation, which promotes neurotoxicity and Aβ accumulation, especially in the cerebral cortex and hippocampal regions, which play important roles in spatial, short-term, and long-term memories. Animal models further confirm Al neurotoxicity, as long-term exposure to AlCl_3_ causes neurological alterations resembling those observed in AD [4]. Aluminum intoxication resulted from prolonged exposure and regular use, including inhalation, consumption of food and water, usage of aluminum cutlery, food additives, medications, deodorants, hemodialysis, and vaccination [5].

Through a variety of multifactorial mechanisms, such as oxidative stress, inflammation, abnormal signaling pathways, and mitochondrial dysfunction, the pathogenic Aβ proteins implicated in AD contribute to neurodegeneration. Together, these elements encourage apoptosis, which damages neurons and ultimately causes death. Amyloid plaques, formed by the BACE1 and γ-secretase-catalyzed proteolytic cleavage of amyloid precursor protein (APP), as well as neurofibrillary tangles (NFTs), consisting of hyperphosphorylated Tau proteins, are two important pathogenic characteristics of AD. In neurons, Aβ aggregation causes severe cytotoxicity, which damages synapses and phosphorylates tau proteins, reinforcing the progression of AD through complex synergistic interactions [1, 5].

Furthermore, a key element in AD is the decreased expression of brain-derived neurotrophic factor (BDNF), which is necessary for neuronal development and memory. The disease advances as a result of the reduction in BDNF levels, which encourages the development of Aβ plaques and NFTs [6, 7]. miR-134 is a brain-specific small non-coding RNA (about 22 nucleotides) that negatively regulates synaptic plasticity by inhibiting the expression of proteins essential for synapse formation. Elevated levels of miR-134 have been associated with impaired synaptic function and cognitive deficits. Because of their regulatory roles and high brain expression, miRNAs are promising therapeutic targets for neuroprotection and recovery in neurological disorders [8]. In AD, miRNAs influence disease progression by modulating Aβ production, clearance, and Tau hyperphosphorylation, which are central to its pathology.

Current treatments for AD alleviate symptoms, but they may have negative side effects and do not stop the disease progression [9]. Consequently, more research is being conducted to develop more effective treatments, using natural compounds such as astaxanthin (AST) that have antioxidant and anti-inflammatory qualities, which is suggested as a possible treatment to lessen neurodegeneration.

Through a variety of mechanisms, AST prevents oxidative damage by scavenging radicals, preventing lipid peroxidation, quenching singlet oxygen, and regulating the expression of genes associated with oxidative stress [10]. The reddish-orange carotenoid astaxanthin is found in a variety of marine animals, such as shrimp and salmonids. Microalgae such as *Haematococcus pluvialis*, *Chlorella vulgaris*, *Chlorella zofingiensis*, and *Chlorococcum* species also produce AST [11]. Research indicates that AST is biologically more active than comparable carotenoids like lutein and zeaxanthin [11]. According to clinical research, AST supplementation lowers biomarkers associated with AD and enhances cognitive performance [12].

Because of its high lipophilicity and poor solubility in gastrointestinal fluids, AST has a limited oral bioavailability despite its advantages [13, 14]. Advanced delivery methods, such as lipid nanoparticles and invasomes, have been created to overcome these obstacles. Invasomes are nano vesicular carriers composed of phospholipids, ethanol, and penetration enhancers, designed to facilitate enhanced systemic drug delivery and improved bioavailability [15, 16]. A possible method for AST administration in the treatment of AD is the use of invasomes, which in particular exhibit remarkable qualities as carriers for both hydrophobic and hydrophilic medications, making them a promising vehicle for AST delivery in AD treatment. Encapsulating AST in invasomes may help overcome the limitations of AST’s poor oral bioavailability and enhance its delivery to the brain, thereby amplifying its antioxidant and neuroprotective effects against AD-related neurodegeneration.

This study was designed to create an astaxanthin-loaded invasome (AST-LI) formulation as a drug delivery method and to evaluate its multi-targeted efficacy on SIRT-1/BDNF/miRNA-134/GSK-3β signaling in rats with an Alzheimer’s model induced by aluminum chloride, by employing various neurochemical changes, behavioral scoring, and histopathological examinations.

## Materials and Methods

### Chemicals

Phospholipid, cineole, cholesterol, chloroform, methanol, and ethanol were obtained from the *Agitech Pharmaceutical Company* (*Cairo*, *Egypt*). Aluminum chloride hexahydrate (AlCl_3_. 6H_2_O) was bought from *Qualikems Fine Chem Pvt*. *Ltd*., *68–69 G.I.D.C*., *Industrial Estate Nandesari*, *Vadodara Pin: 391,340* (*India*), with MW = 241.43 g/mole, purity ≥ 98.0%, and CAS number 7784–13-6. Astaxanthin (AST) (C40H52O4) was purchased from *Acros Organics*, *Thermo Fisher Scientific*, *Janssen Pharmaceuticalaan 3a*, *2440 Geel*, *Belgium*. Its MW = 596.85 (g/mol), purity ≥ 98.0%, and CAS Number: 472–61-07. All of the chemicals used in this experiment, unless otherwise noted, were purchased from *Sigma-Aldrich in St*. *Louis*, *Missouri*, *USA*.

### Preparation of AST-Loaded Invasome (AST-LI)

The thin-film hydration method was used to develop AST-LI formulations [17]. A thin layer of invasomes was formed by dissolving AST (10 mg), phospholipid (300 mg), cineole (0.1 ml), and cholesterol (16 mg) in an organic solvent solution of chloroform and methanol. The mixture was then vaporized using a rotary evaporator under vacuum at 40 °C and 100 rpm. The consequential suspension was kept at 4 °C after the invasomal film was rehydrated at 60 rpm using a phosphate buffer and ethanol (0.3 ml) solution.

### Characterization of AST-Loaded Invasomes

#### Zeta Potential (ZP) and Particle Size (PS)

Using a Zeta Sizer device (Malvern, Worcestershire, UK), the mean of ZP, PDI, and PS of the prepared AST-LI was determined using dynamic light scattering. ZP is used to assess the stability of AST-LI depending on repulsive and attractive forces among vesicles. PDI was employed to quantify the homogeneity of vesicle size and distribution within a sample. The vesicle size was assessed in triplicates using a diluted sample of AST-LI formulations [18].

#### Differential Scanning Calorimetry (DSC)

The Shimadzu DSC-50 (Shimadzu Corporation, Kyoto, Japan) was used in order to assess the AST thermograms, cholesterol, phospholipid, and AST-LI in order to look at the relationship between AST and the components that produce invasomal formation [19]. The DSC scan was obtained with a constant nitrogen flow rate of 100 ml/min throughout a temperature range of 25 to 300 °C at a steady 10 °C/min.

#### Fourier-Transform Infrared Spectroscopy (FT-IR)

FTIR (8400 s, Shimadzu, Japan) was used to examine the chemical interactions of AST, cholesterol, phospholipid, and AST-LI [20]. Before the samples were analyzed from 4000 to 400 cm^−1^, they were completely ground and combined with KBr.

#### Transmission Electron Microscopy (TEM)

By the use of TEM (JEM-1230, Jeol, Tokyo, Japan), the morphology of AST-LI was obtained. A sample of AST-LI formulations is diluted, placed on the carbon grid, and then colored with 2% phosphotungstic acid (PTA) as described in the study of Salem [21].

### Experimental Animals

Twenty-eight fully grown male albino rats, weighing 120–150 g, were obtained from the Helwan farm of research animals located in Cairo, Egypt. After 2 weeks of acclimatization, the rats were kept in groups in metal cages with adequate ventilation and lighting within a 12-h light-dark cycle at room temperature (24 °C ± 2 °C) and humidity (68%) for the duration of the experiment. The rats were provided with unlimited access to food and water. The Institutional Animal Care and Use Committee at Beni-Suef University authorized all experimental procedures, which were carried out rigorously in compliance with the rules governing the handling and care of lab animals. The procedures were approved under approval number 024-017.

### Experimental design

#### Induction of Alzheimer’s Disease (AD)

**Scheme 1:**
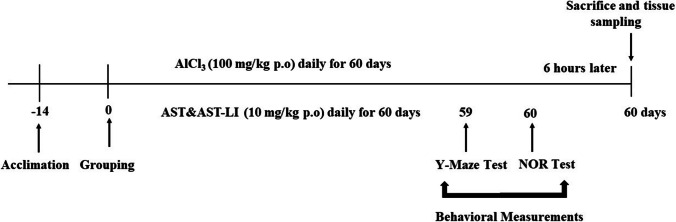
Scheme describing the experimental protocol used in this study

Animals were given 0.5 ml of the hydrated AlCl_3_ (AlCl_3_.H_2_O) solution dissolved in distilled water by oral gavage for 60 days at a dose of 100 mg/kg/day to generate the AD model [22]. The rats were divided into four equal groups (*n* = 7 each) at random in the manner described below (Scheme 1):

i. Control group: The rats were daily orally gavaged with 0.5 ml/kg b.w of distilled water only (vehicle).
ii. AlCl_3_ group: The rats were orally gavaged with 0.5 ml freshly prepared hydrated aluminum chloride miscible in distilled water (100 mg/kg b.w/day) for 60 days.
iii. AST + AlCl_3_ group: Each rat received a daily oral gavage of AST (10 mg/kg b.w) 1 h prior to the administration of AlCl_3_ [14].
iv. AST-LI + AlCl_3_ group: Every day, each rat was given an oral gavage of AST-LI (10 mg/kg b.w) 1 h prior to the administration of AlCl_3._

### Behavioral Measurements

At the last 2 days of the experiment, Y-maze and novel object recognition maze were used to evaluate behavioral alterations of memory and cognition of the tested rats. Five rats were used in the Y-maze test. After 24 h, they were placed in the novel recognition maze. Each maze was cleaned and wiped with alcohol 70% before placing each rat (Scheme 1).

#### Y-Maze Test

This test was conducted 1 day before the end of the experiment to evaluate the impairment of spatial short-term working memory [23] as an indication of the induction of Alzheimer disease (Scheme 1). The maze is made of wooden black-painted 3 arms (A, B, and C). Each arm of the Y-maze was 40 cm long, 30 cm high, and 15 cm wide as described by Rasoolijazi et al. [24]. Each rat was placed in the maze at the end of a fixed arm (A) and left for 8 min to move freely between the three arms. The sequence of each arm entry was recorded manually (i.e., ACBABACACBCAC AC, etc.). Spontaneous alteration behavior percent (SAP %) was calculated according to Rasoolijazi et al. [24].

$$(SAP\%)=Actual\;alteration/maximum\;alteration\;(total\;arm\;enteries-2)\times100$$

#### Novel Object Recognition (NOR) Test

This test was performed at the last day of the experiment, 24 h after the Y-maze test and 6 h before tissue sampling, to allow recovery after behavioral measurement handling stress (Scheme 1). The NOR maze was designated to assess the impairment in memory and learning in the rats depending on the recognition memory following Bevins and Besheer [25], Leger et al. [26], and Lim et al. [27] with little modification. The maze consists of a square (40 × 40 cm) arena 60 cm in height. The test condition was 2 phases for each rat. In the first (familiarization or training) phase, each rat was allowed to explore and identify 2 objects for 10 min. One hour later, the test phase was conducted where the rat was exposed to the 2 familiar objects and another novel one for 5 min. In the test phase, the exploration of the rats to the objects was videotaped using a digital camera. Then after, the videos were displayed for recording the number of attempts and time of exploring the familiar and novel objects to calculate the discriminating index (DI) and discriminating ratio (DR) parameters. Exploration was scored only when rats directed its nose 2 cm or less towards the object.

$$\begin{array}{c}\begin{array}{c}DI=\frac{Time\;exploring\;novel\;object}{Total\;time\;exploring\;both\;novel\;and\;familiar\;objects}\\DI=\frac{Number\;of\;attempts\;to\;explore\;novel\;object}{Total\;Number\;of\;attempts\;to\;explore\;both\;novel\;and\;familiar\;objects}\end{array}\end{array}$$

### Sampling and Tissue Preparations

Animals were sacrificed by cervical dislocation at the completion of the experiment. The brains were removed, cleansed, and washed using physiological saline (0.9% sodium chloride) after the incision in the dorsal side of the skull. One hemisphere’s cortex and hippocampus were excised in order to prepare a tissue homogenate and extract all of the RNA needed for qPCR. For histological inspection, the opposite hemisphere was used. Using a homogenizer (Ortoalresa, Spain), 5 ml of phosphate-buffered saline was used to homogenize the first portion (pH: 7). For further biochemical tests of reduced glutathione (GSH), malondialdehyde (MDA), and catalase activity, the supernatant was stored at − 20 °C after being centrifuged at 20,000 × g for 15 min at 4 °C. In order to determine the gene expression, the second part of the brain tissue was kept at − 80 °C. For histological analysis, the third piece of brain tissue was rinsed with saline before being immersed in 10% neutral buffered formalin.

### Biochemical Assays

#### Determination of Oxidative Stress Markers

Glutathione (GSH) was assessed in brain tissue homogenates using the techniques outlined by Beutler and Kelly [28]. GSH reduces DTNB (5,5′-dithiobis(2-nitrobenzoic acid) to form a yellow-colored product. The absorbance of this product, measured at 405 nm, is directly proportional to the GSH concentration. The brain concentration of MDA was quantified using the method of Satoh [29], which relies on the interaction of thiobarbituric acid with malondialdehyde in an acidic medium. The absorbance of the resulting pink product can be measured at 534 nm, and MDA concentrations were calculated from a standard curve. Catalase activity is measured in brain tissue homogenate using the method outlined by Aebi [30], which involves catalase reacting with a known amount of hydrogen peroxide (H₂O₂). After 1 min, the process is stopped with catalase inhibitors. The residual H₂O₂ combines with DHBS (3,5-dichloro–2–hydroxyl benzene sulfonic acid) and 4-aminophenazone (AAP) in the presence of peroxidase (HRP) to generate a colored dye. Color intensity is inversely proportional to catalase activity. The Biodiagnostic Company for Research Kits in Egypt provided the commercial kits for determination of GSH, MDA, and catalase.

#### Assays of Rat Amyloid Beta (Aβ1-42), BDNF, GFAP, CX3CL1, GSK-3β, SIRT-1, and Serotonin Contents

Following the manufacturer’s instructions, Aβ1-42, GSK-3β, BDNF, GFAP, fractalkine (CX3CL1), SIRT-1, and serotonin rat enzyme linked immunosorbent assay (ELISA) kits were used to measure the cortical and hippocampus contents of these substances, respectively. The necessary ELISA kit was used to assess the levels of amyloid β-protein (Aβ) in accordance with the manufacturer’s instructions (*Biorbyt*, *LLC. San Francisco*, *California 94,104*, *USA*). *The BDNF ELISA kit* (*biosensis, Pty Ltd*, *Thebarton 5031*, *SA*, *Australia*) was used to measure the BDNF concentrations. The glial fibrillary acidic protein (GFAP) ELISA kit (*Elabscience*, *Houston*, *Texas*, *77,079*, *USA*) and chemokine C-X3-C-motif ligand 1 (CX3CL1) ELISA kit (*Fine Test*, *Wuhan*, *Hubei*, *430,206*, *China*) were used to measure the concentrations of GFAP and CX3CL1, respectively. Using a GSK-3β ELISA kit (*Fine Test*, *Wuhan*, *Hubei*, *430,074*, *China*), GSK-3β concentrations were ascertained. The SIRT-1 ELISA kit (*Bioss*, *Inc*., *Woburn*, *Massachusetts 01801*, *USA*) was used to measure SIRT-1 concentrations. The Serotonin ELISA kit (*Enzo Life Sciences Inc*., *10 Executive Boulevard*, *Farmingdale*, *NY 11735*) was used to measure the levels of serotonin.

#### Estimation of p-Tau by Western Immunoblotting

Each sample of brain tissue homogenate for each group was supplemented with the ReadyPrepTM protein extraction (total protein) kit from Bio-Rad Inc (Catalog #163–2086) in accordance with the manufacturer’s instructions. The Bradford Protein Assay Kit (SK3041) was supplied by Bio basic Inc (Markham Ontario L3R 8T4 Canada) for quantitative protein investigation [31]. After being separated using 10% SDS–polyacrylamide gel electrophoresis, 20 μg of protein per lane was transferred onto polyvinylidene difluoride (PVDF) membranes. After being incubated for 2 h with the Tris-buffered saline (TBS) (10 mM of Tris–Cl, 100 mM of NaCl, pH = 7.5) which includes 5% nonfat-dried milk and 0.1% Tween 20, the membrane was tested as a loading control using 1^ry^ antibodies to p-Tau and beta-actin. Membrane development and visualization by means of the Amersham detection kit and chemiluminescence is directed by the manufacturer. Using image analysis software and protein normalization on the ChemiDoc MP imager, the band intensities of the target proteins were compared to those of the control sample, beta-actin. The 1^ry^ and 2^ry^ antibodies were bought from Cell Signaling Technologies. β-Actin is employed as a reference control.

#### Determination of miRNA-134, BAX, BCL2, Caspase 3, MAO, and AChE Genes Expression

Following the manufacturer’s instructions, RNA was extracted from tissue lysate using the Direct-zol RNA Miniprep Plus (Cat# R2072, ZYMO RESEARCH CORP. USA) and the Reagent of TRIzol (Life Technologies, USA). Then, by the use of SuperScript IV One-Step RT-PCR kit (Cat# 12,594,100, Thermo Fisher Scientific, Waltham, MA USA), the isolated RNA was reverse-transcribed and then the PCR was achieved in a single step. Table 1 contains the gene-specific primers. The primers were proposed using the NCBI platform. Forty cycles made up the thermal cycling profile, which included 10 s of denaturation at 95 °C, 15 s of annealing at 58 °C, and 15 s of extension at 72 °C [32]. A melting curve was then employed to verify specificity. The ΔΔCt was used to estimate the relative quantification of each gene expression. The gene expression data were standardized using housekeeping genes (U6) for *miRNA-134* and β-actin for other mRNA genes [33]. There are no template controls for any gene in any experiment. Double analyses were performed on each sample [34]. Each target gene’s relative quantitation (RQ) is measured using the delta-delta Ct (ΔΔCt) computation. Using 2^−∆∆Ct^, we determined each gene’s RQ [35].

**Table 1:** The primer set of the considered genes

	**Sense**	**Antisense**	**Amplicon**	**Accession no**
*miRNA134*	TGTTTCCTCATGACTGCCCC	CGATCCGGGTTTCCGTGTTA	121	XR_005489062.2
*U6*	GCTTCGGCAGCACATATACTAAAAT	CGCTTCACGAATTTGCGTGTCAT	165	XR_010061656.1
*Casp3*	GAGCTTGGAACGCGAAGAAA	TTGCGAGCTGACATTCCAGT	221	NM_012922.2
*BAX*	CACGTCTGCGGGGAGTCAC	TTCTTGGTGGATGCGTCCTG	248	NM_017059.2
*BCL-2*	TCGCGACTTTGCAGAGATGT	CAATCCTCCCCCAGTTCACC	116	NM_016993.2
*AChE*	AGGACGAGGGCTCCTACTTT	CATGGCATCTCTCAGGTGGG	200	NM_172009.1
*MAO*	GTGCCTGGTCTGCTCAAGAT	GGCCCAAACCATAGGCTGTA	168	NM_033653.1
*β-actin*	CCGCGAGTACAACCTTCTTG	CAGTTGGTGACAATGCCGTG	297	NM_031144.3

### Histopathological Examination

The cortex and hippocampus of one hemisphere were dehydrated by immersing in increasing concentrations of ethyl alcohol, then cleaned and clarified in xylene, impregnated in soft paraffin, imbedded in hard paraffin, and sectioned using a rotating microtome to a thickness of 4–6 µm; after that, the tissues were placed on clear and dry glass slides. Several histopathological stains were used for tissue examination:

1. Hematoxylin and eosin as general
2. Congo red stain for detection of amyloid in brain tissue
3. Luxol fast blue for myelin sheath detection.

A light microscope fitted to a LEICA (DFC290 HD system digital camera, Heerbrugg, Switzerland) with 10, 20, and 40 objective lenses was used to examine and observe the acquired slides [36].

### Statistical Analysis

SPSS (version 27.0.1) was used to statistically analyze the results. The experimental groups were compared using the Tukey’s post hoc test after the one-way analysis of variance (ANOVA) test. Spontaneous alteration behavior percent was analyzed using the Kruskal–Wallis test. The data were statistically significant at the *p* < 0.05 level and were displayed as the mean with standard error of the mean.

## Results

### *In vitro *Characterization of AST-Loaded Invasome Formulations

#### Experimental Scheme

In this study, the Box-Behnken design was used to successfully prepare and optimize AST-loaded invasomes. Prior to formulation, studies demonstrated that increasing the phospholipid content improved the entrapment efficiency and vesicle size. For example, when the concentration of phospholipid was increased from 100 to 300 mg, the vesicle size increased from 158.3 ± 5.07 to 420.9 ± 8.67 nm. But the vesicle size grew from 420.9 ± 8.67 to 634.1 ± 14.56 nm when the concentration of phospholipid was increased from 300 to 500. Pre-formulation research was conducted in light of these findings, and a formulation of an AST-LI containing 300 mg of phospholipid was created. Prior to formulation, research was done to determine the amounts of cineole and ethanol. Pre-formulation experiments showed that entrapment efficiency declined with increasing ethanol content; moreover, entrapment efficiency reduced from 87.37 ± 0.68 to 79.87 ± 0.58% when the concentration of ethanol increased from 0.3 to 0.5 ml. Pre-formulation experiments displayed that the cineole concentration had a beneficial effect on the vesicle size; however, as the cineole content increased from 0.1 to 0.2 ml, the vesicle size fluctuated from 240.5 ± 7.4 to 315.14 ± 8.64 nm. Consequently, a combination of cineole (0.1 ml), ethanol (0.3 ml), and phospholipid (300 mg) was used to create invasomes.

### Characterization of the Optimized AST-Loaded Invasomes

#### Zeta Potential and Size Distribution

**Fig. 1:**
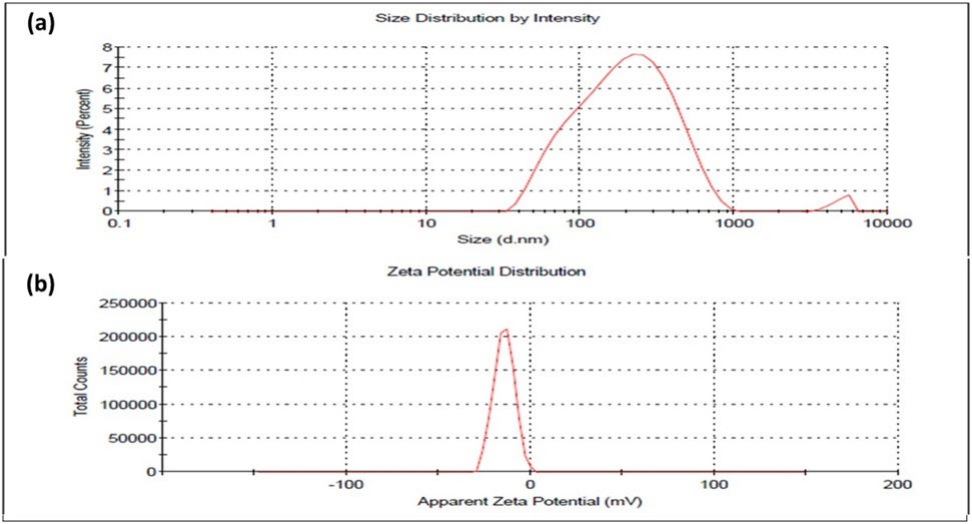
**a** Vesicle size and **b** zeta potential of AST-loaded invasomes. AST, astaxanthin

Figure 1a shows the size distribution of the invasome formulations loaded with AST. An ideal PDI of 0.386 indicates a homogeneous vesicle population and a formulation that is significantly monodispersed. The zeta potential of the AST-LI formulation is shown in Fig. 1b. According to the results, the zeta potential was (−) 14.4, indicating both their physical stability and de-aggregation ability.

#### Differential Scanning Calorimetry (DSC)

**Fig. 2:**
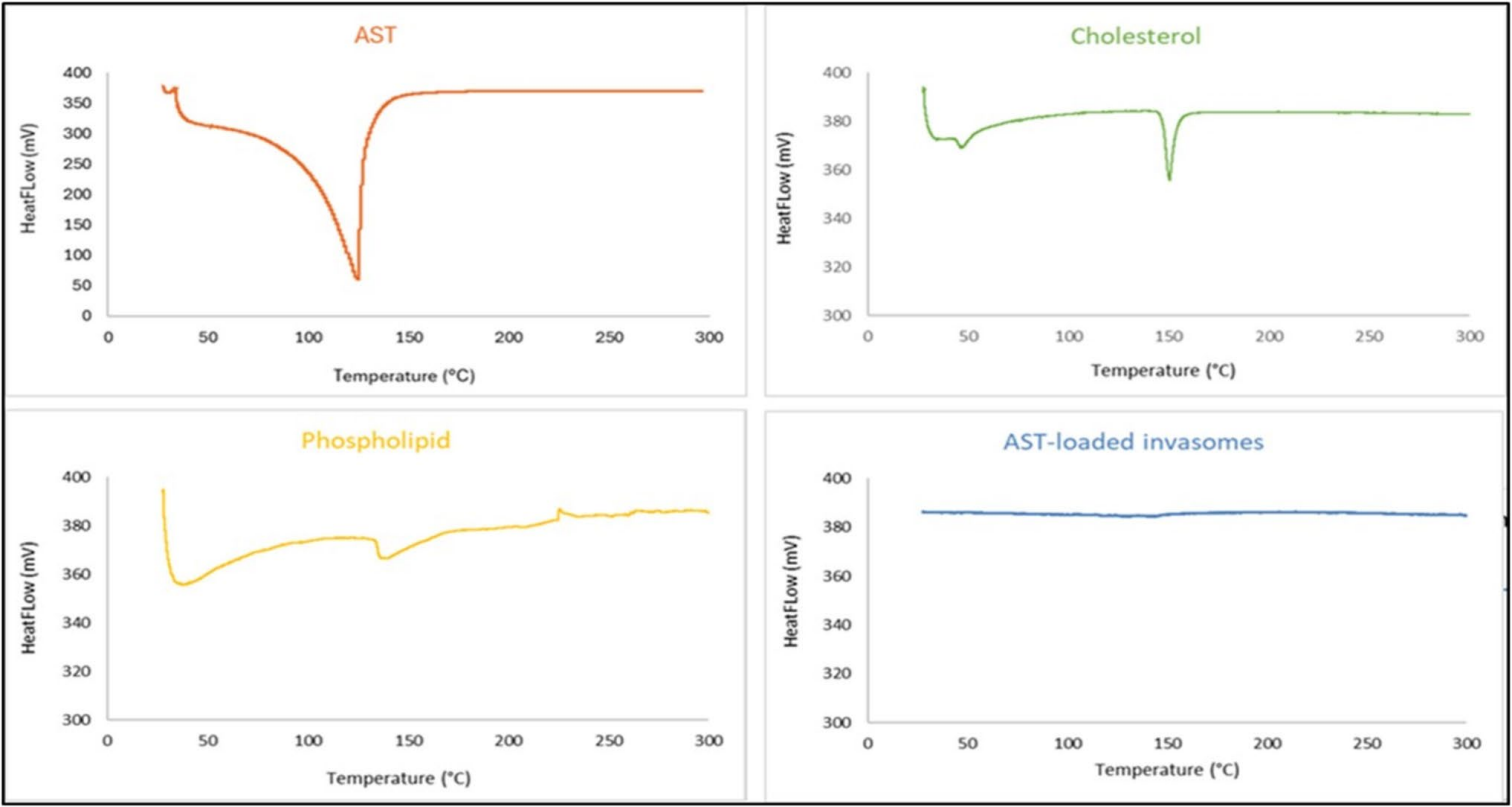
DSC thermograms of AST-loaded invasomes. DSC, differential scanning calorimetry; AST, astaxanthin

The AST thermogram showed an endothermic peak at 125.65 °C, which is also its melting temperature, as seen in Fig. 2. The phospholipid thermogram displayed a prominent endothermic peak at 134.96 °C, which is also its melting point. At 153.35 °C, the melting point of cholesterol, an endothermic peak was visible on the thermogram. These peaks vanished from the AST-LI thermogram. The DSC of AST-LI formulations did not show the endothermic peak of AST, indicating that AST was amorphous and fully integrated following invasome preparation. The results of the DSC analysis of the AST-LI formulations indicate that the lipid bilayer entraps AST more efficiently.

#### Fourier-Transform Infrared Spectroscopy (FTIR)

**Fig. 3:**
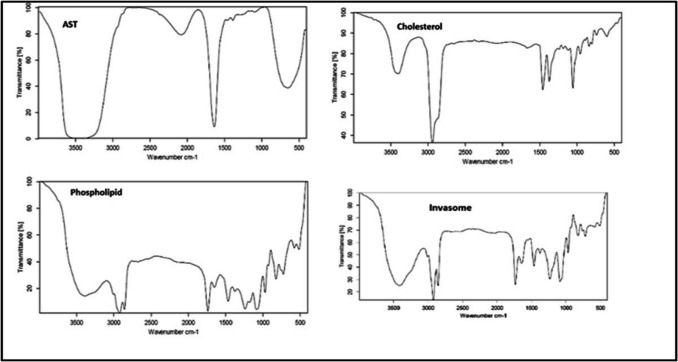
FTIR spectrums for the AST-loaded invasomes. FTIR, Fourier-transform infrared spectroscopy; AST, astaxanthin

The FTIR spectra of AST, phospholipid, cholesterol, and AST-LI are shown in Fig. 3. Phospholipid FTIR spectra revealed peaks for the symmetrical C = O stretching vibration at 1738 cm^−1^, the PO_4_ antisymmetric stretching bands at 1239 cm^−1^, and the CH_2_ stretching vibration at 2924 and 2857 cm^−1^. The cholesterol FTIR spectrum showed peaks for the OH stretching group at 3399 cm^−1^, the CH_2_ stretching vibration at 2940 cm^−1^, the double bond C = C at 1670 cm^−1^, and the asymmetric CH_2_ stretching vibrations at 1459 cm^−1^. Peaks for the amide I of proteins and the C = O band were visible in the AST FTIR spectra at 1638 cm^−1^ and 1095 cm^−1^, respectively, for the C–O–C in carbohydrates. The FTIR spectra of the AST-LI showed similar peaks, suggesting that the formulation’s constituent parts were compatible.

#### Transmission Electron Microscopy (TEM)

**Fig. 4:**
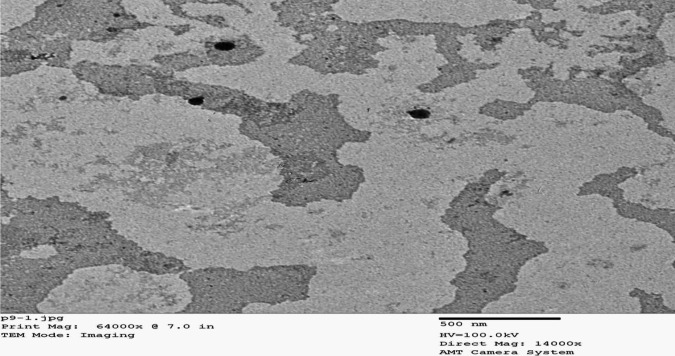
TEM image of AST-loaded invasomes. TEM, transmission electron microscopy; AST, astaxanthin

TEM micrographs (Fig. 4) revealed the development of spherical vesicles with a distinct core and contour that were free of aggregates.

### *In vivo *Characterization of AST-Loaded Invasome Formulations

#### Impact of AST and AST-LI on Learning, Memory, and Cognition of Rats

**Fig. 5:**
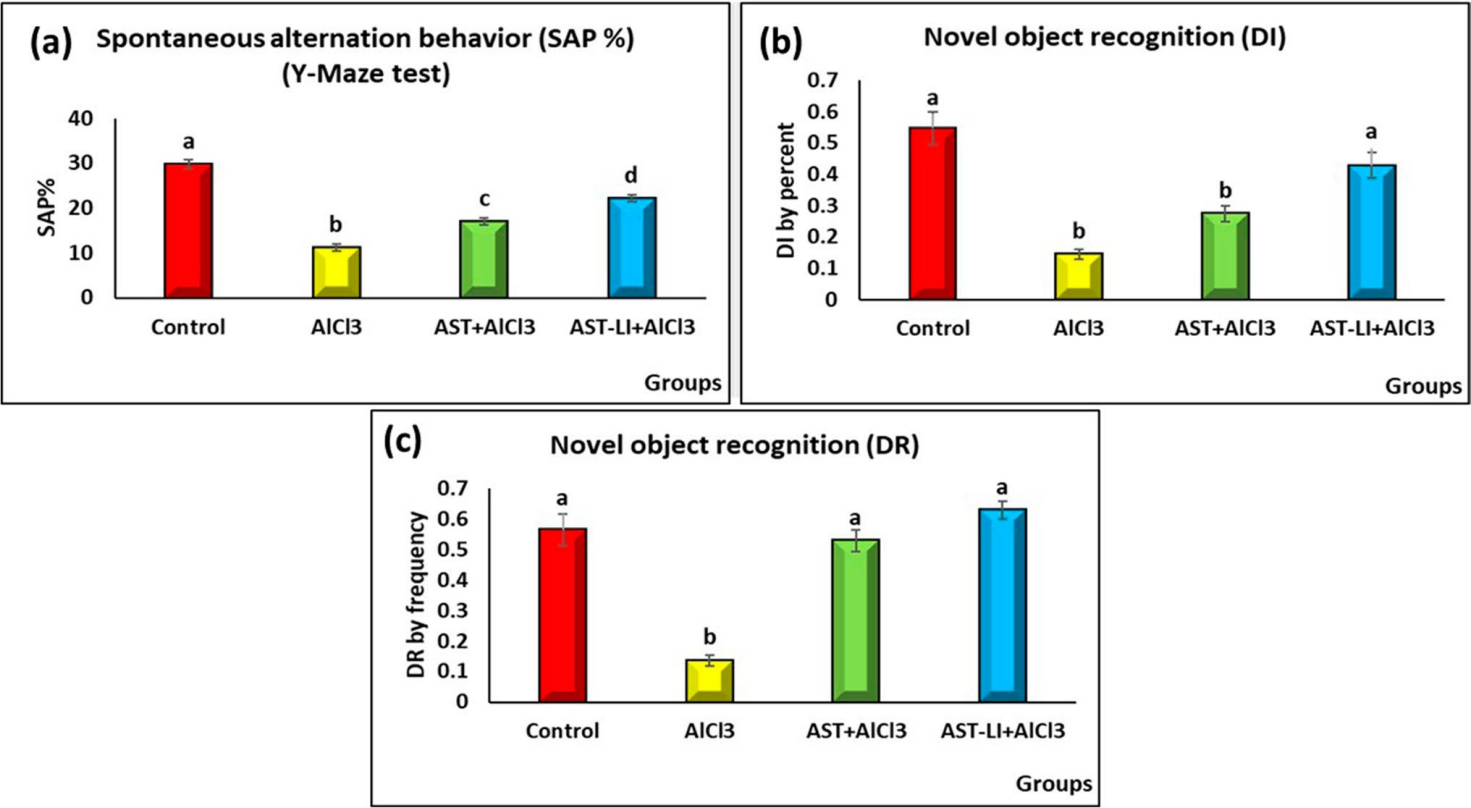
Effect of AST and AST-LI on **a** SAP%, **b** DI, and **c** DR activity in the brain tissues of rats in different groups. Values are represented as mean ± standard error of mean (*n* = 7). Columns with different superscript letters are significantly different at *p* < 0.05. AlCl3, aluminum chloride; AST-LI, astaxanthin-loaded invasomes; SAP, spontaneous alternation behavior; DI, discrimination index; DR, discrimination ratio

Figure 5 shows the changes in spatial short-term memory (SAP %) and memory cognition (DI and DR). The AD-like untreated rats displayed significantly lower values (*p* < 0.05) of SAP, DI, and DR than the control group. The AST and AST-LI groups had significantly (*p* < 0.05) higher scores of SAP, DI, and DR than AD-like rats. On the other hand, SAP and DI were significantly increased (*p* < 0.05) in AST-LI formulations as compared with the AST group, while in DR, there is a non-significant increase in AST-LI formulations.

#### Effect of AST and AST-LI on AChE, MAO and Serotonin Levels in Rat Brains in Different Groups

**Fig. 6:**
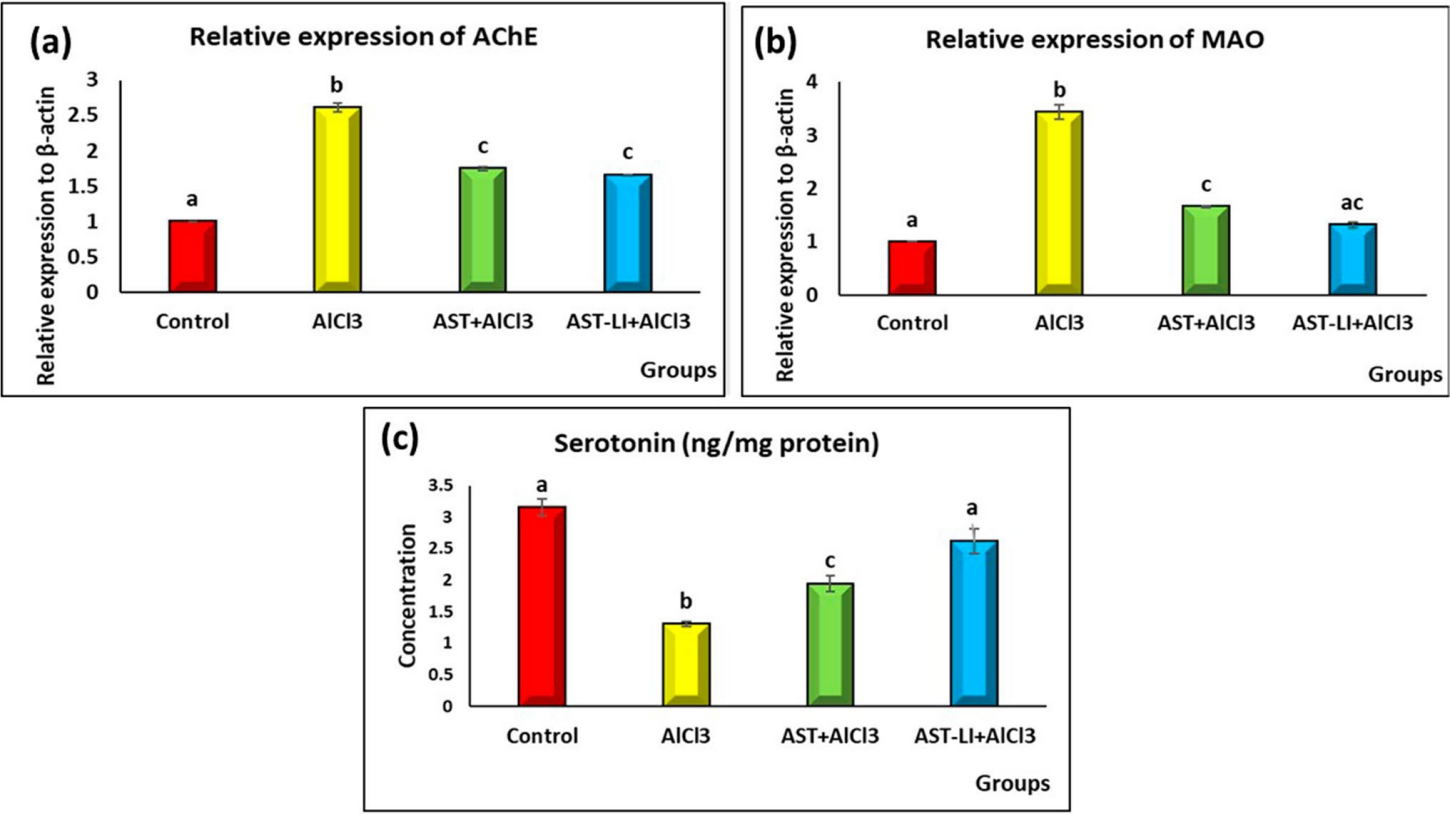
Effect of AST and AST-LI on **a** AChE, **b** MAO, and **c** serotonin activity in the brain tissues of rats in different groups. Values are represented as mean ± standard error of mean (*n* = 7). Columns with different superscript letters are significantly different at *p* < 0.05. AlCl3, aluminum chloride; AST-LI, astaxanthin-loaded invasomes; MAO, monoamine oxidase-A; AChE, acetylcholinesterase enzyme

The results in Fig. 6 revealed a significant increase in AChE and MAO mRNA expression levels and a significant decrease (*p* < 0.05) in serotonin levels in the AD-untreated rats compared to the control ones. Treatment with AST and AST-LI formulations demonstrated a considerable ameliorative effect by restoring the values to almost normal.

#### Effect of AST and AST-LI on Brain Redox Markers in Different Groups

**Fig. 7:**
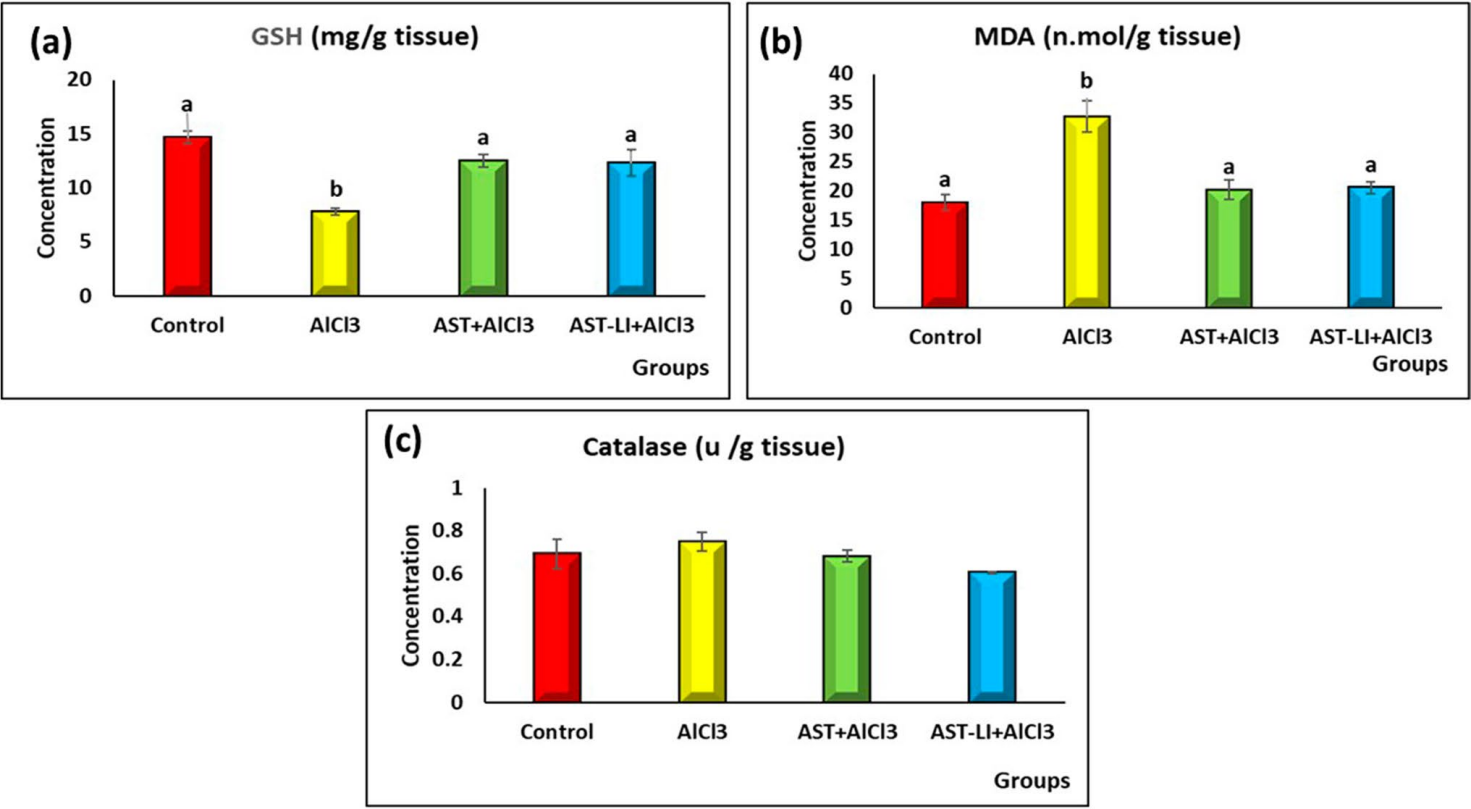
Effect of AST and AST-LI on **a** GSH, **b** MDA, and **c** catalase activity in the brain tissues of rats in different groups. Values are represented as mean ± standard error of mean (*n* = 7). Columns with different superscript letters are significantly different at *p* < 0.05. AlCl3, aluminum chloride; AST-LI, astaxanthin-loaded invasomes; GSH, reduced glutathione; MDA, malondialdehyde

As illustrated in Fig. 7, the AD-like untreated rats showed a significant decrease (*p* < 0.05) in GSH and a significant increase in MDA concentrations (*p* < 0.05) in comparison to control rats, but the activity of catalase enzyme in brain tissue homogenates was non-significantly changed among different rat groups. Treatment with AST and AST-LI formulations significantly showed an ameliorator impact by returning the results to almost normal.

#### Effect of AST and AST-LI on Brain Aβ1-42, p-Tau, GFAP, and CX3CL1 Concentrations in Different Groups

**Fig. 8:**
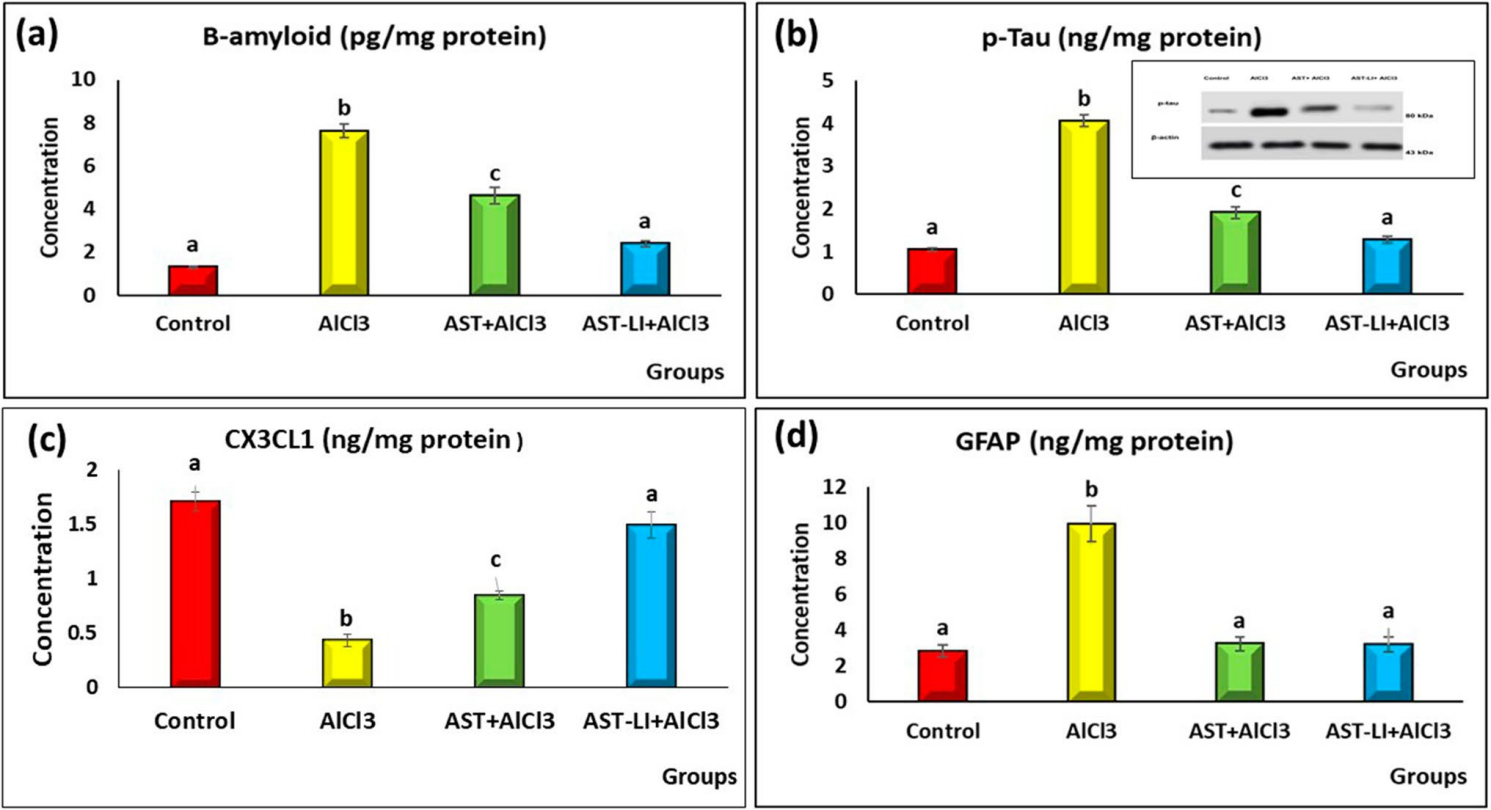
Effect of AST and AST-LI on **a** β-amyloid, **b** p-Tau, **c** CX3CL1, and **d** GFAP concentrations in the brain tissues of rats in different groups. Values are represented as mean ± standard error of mean (*n* = 7). Columns with different superscript letters are significantly different at *p* < 0.05. AlCl3, aluminum chloride; AST-LI, astaxanthin-loaded invasomes; p-Tau, phosphorylated tau protein; CX3CL1, chemokine C-X3-C-motif ligand 1; GFAP, glial fibrillary acidic protein

The AD-like untreated groups showed significantly increased (*p* < 0.05) levels of Aβ1-42, p-Tau, and GFAP, and decreased levels of CX3CL1 in brain tissues as compared to the control group. These levels significantly ameliorated in the rats treated with AST and AST-LI in comparison to the AD-like group. The nano-formulations, however, demonstrated the strongest protection when compared to the free form (Fig. 8).

#### Effect of AST and AST-LI on Levels of Brain SIRT-1, GSK-3β, BDNF, and miRNA-134 in Different Groups

**Fig. 9:**
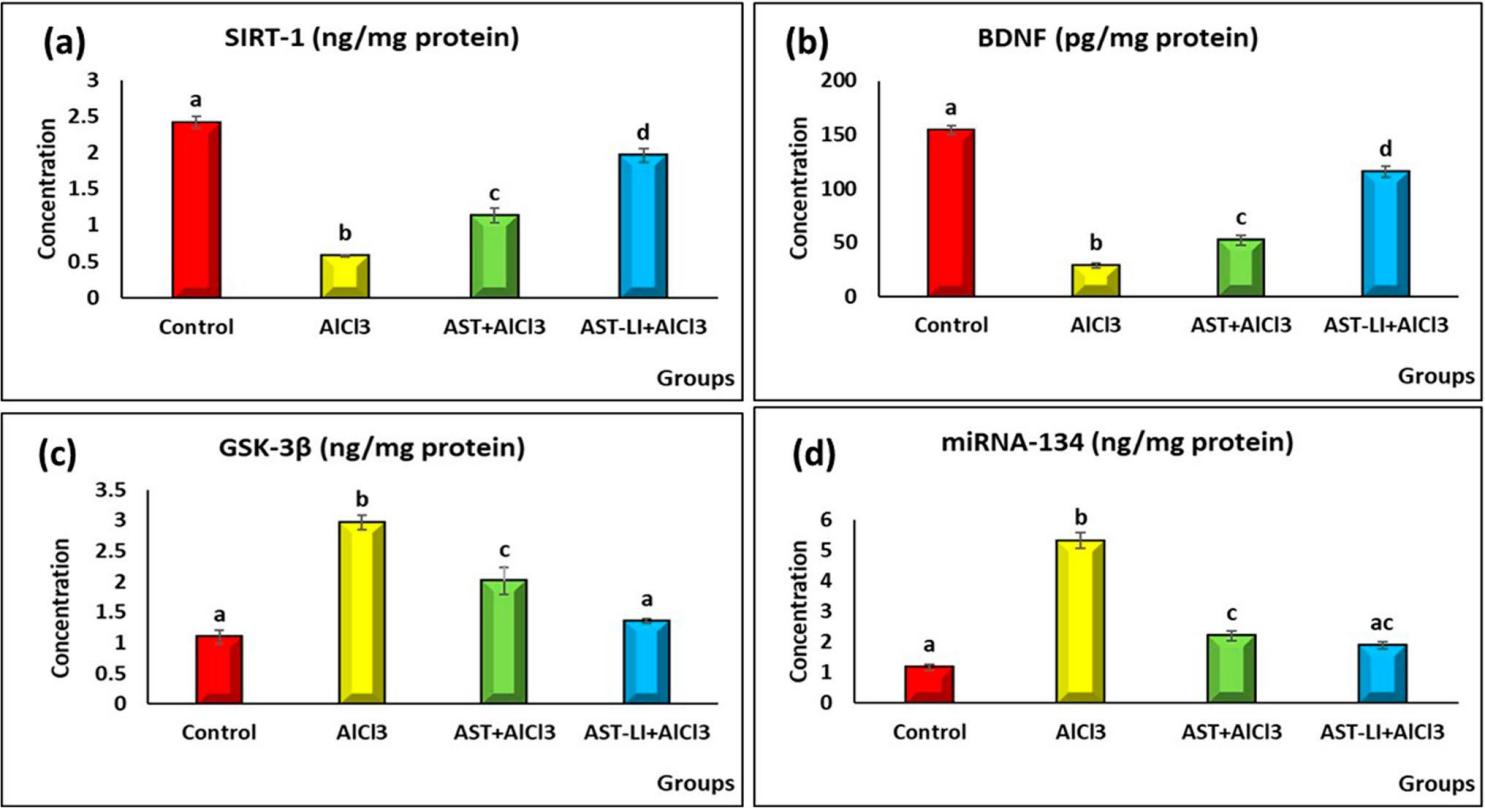
Effect of AST and AST-LI on **a** SIRT-1, **b** BDNF, **c** GSK-3β, and **d** miRNA-134 in the brain tissues of rats in different groups. Values are represented as mean ± standard error of mean (*n* = 7). Columns with different superscript letters are significantly different at *p* < 0.05. AlCl3, aluminum chloride; AST-LI, astaxanthin-loaded invasomes; SIRT-1, Rat Sirtuin 1; BDNF, brain-derived neurotrophic factor; GSK-3ß, glycogen synthase kinase-3 beta; miRNA, micro-ribonucleic acid

The results in Fig. 9 showed that the AD-like untreated group had significantly low levels of SIRT-1 and BDNF (*p* < 0.05) and high levels of GSK-3β and miRNA-134 in brain tissues as compared to the control group. Treatment with AST and AST-LI formulations significantly restored the values. Furthermore, AST-LI formulations achieved the best findings.

#### Effect of AST and AST-LI on Brain BAX, BCL2, BAX/BCL2 Ratio, and Caspase-3 in Different Groups

**Fig. 10:**
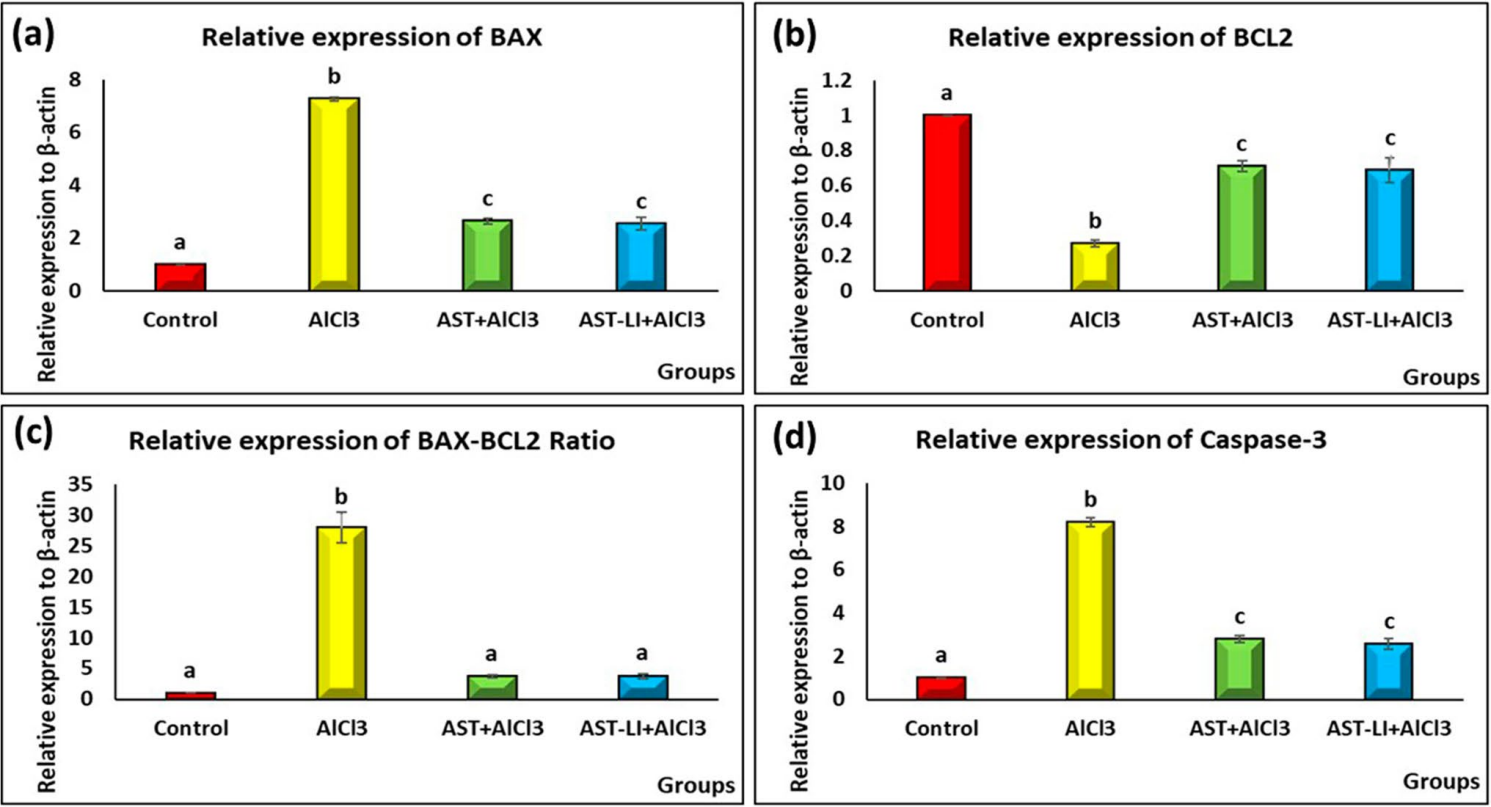
Effect of AST and AST-LI on **a** BAX, **b** BCL2, **c** BAX-BCL2 ratio, and **d** caspase-3 activity in the brain tissues of rats in different groups. Values are represented as mean ± standard error of mean (*n* = 7). Columns with different superscript letters are significantly different at *p* < 0.05. AlCl3, aluminum chloride; AST-LI, astaxanthin-loaded invasomes; BAX, Bcl-2-associated protein x; BCL2, B cell leukemia lymphoma-2 protein; caspase, cysteine aspartic acid-specific protease

As demonstrated in Fig. 10, the AD-untreated group showed significant (*p* < 0.05) higher levels of BAX, BAX/BCL_2_ ratio, and caspase-3, while it showed significant (*p* < 0.05) lower levels of BCL_2_ than the normal control group. The rats treated with AST and AST-LI exhibited an ameliorative effect compared to the AD-treated group.

### Histopathological Findings in the Rats’ Brains in Different Groups

**Fig. 11:**
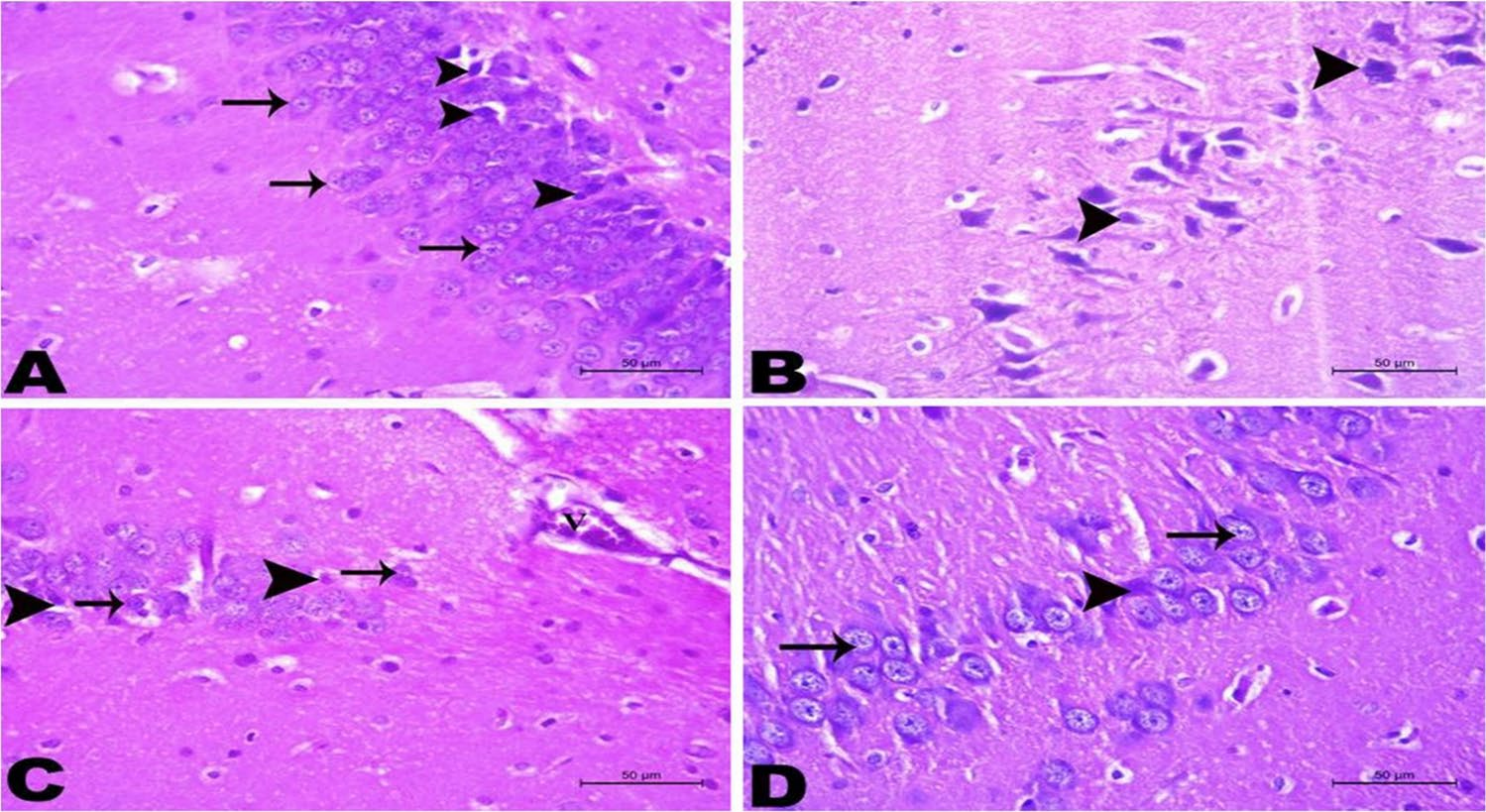
Histopathological changes of the hippocampus in different studied groups. **A** Hippocampus of the normal control group showing normal orientation of the hippocampal tissue containing several layers of normal nerve cells (arrow) and normal neuroglia cells (arrowhead). **B** Brain tissues of AD-like untreated group showing the hippocampal tissue appeared with disrupted cell layers. The neurons (arrow) and neuroglia cells (arrowhead) suffered from degenerative changes and shrinkage. The brain’s vessels (V) appeared highly congested. **C** Brain tissues of AST group, the hippocampal tissue appeared with few cell layers. The neurons (arrow) and neuroglia cells (arrowhead) suffered from mild degeneration. The brain’s vessels (V) showed mild congestion. **D** Brain tissues of AST-LI formulation group showing that the hippocampal tissue accepts its layer organization. The majority of nerve cells (arrow) and neuroglial tissue appeared normal except few cells suffered from degeneration (arrowhead). H&E stain × 400

**Fig. 12:**
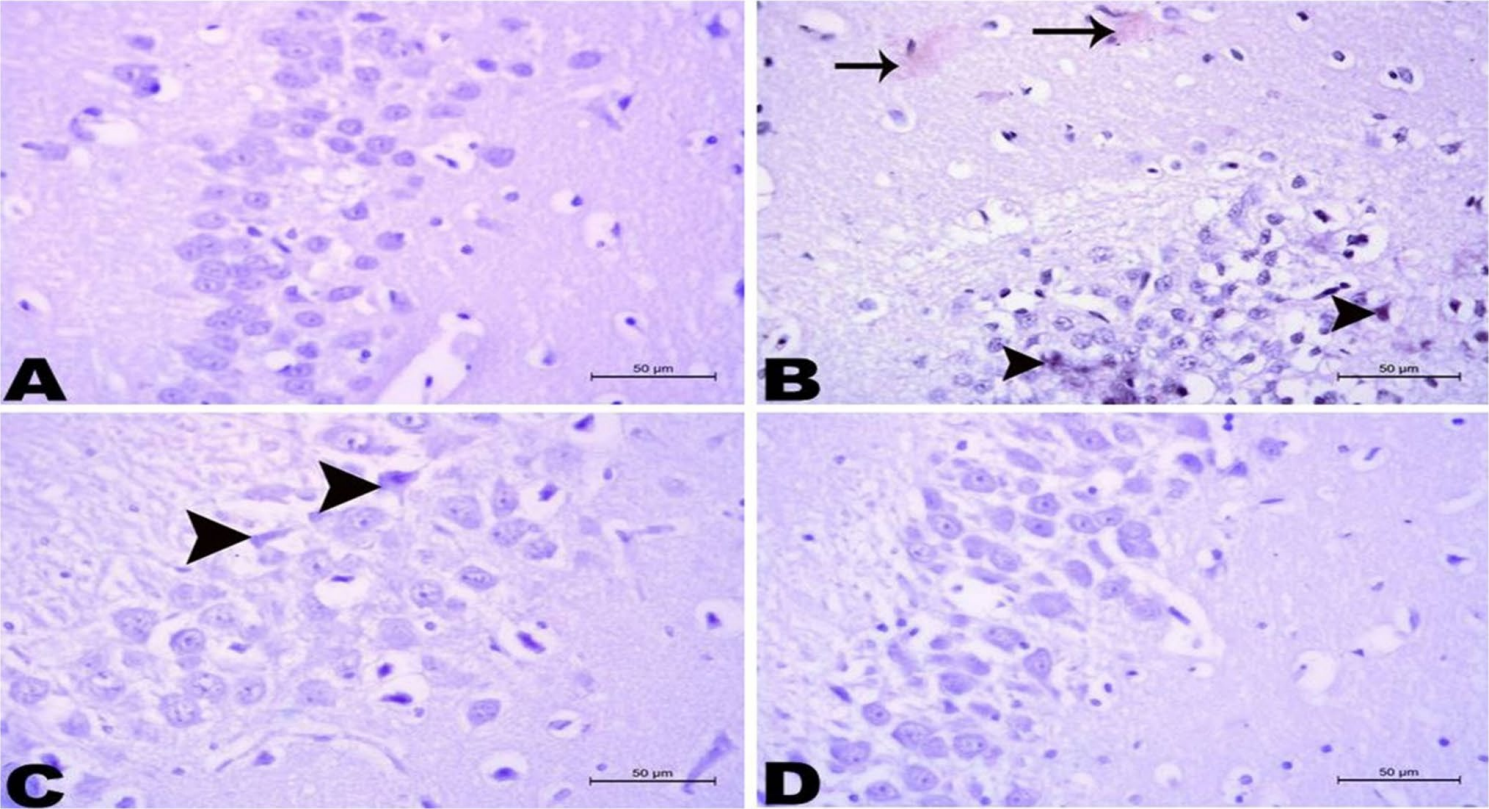
Histopathological changes of brain tissue in different studied groups. **A** Hippocampus of the normal control group did not show any deposition of amyloid deposits. **B** Brain tissues of AD-like untreated group showing accumulation of acidophilic amyloid materials in the hippocampal tissue (arrow) and inside the neurons and neuroglia cells (arrowhead). **C** Brain tissues of astaxanthin (AST) group showing minimal appearance of acidophilic amyloid materials inside the neurons and/or neuroglia cells (arrowhead). **D** Brain tissues of AST-LI formulation group showed no deposition of amyloid deposits. Congo red stain × 400

**Fig. 13:**
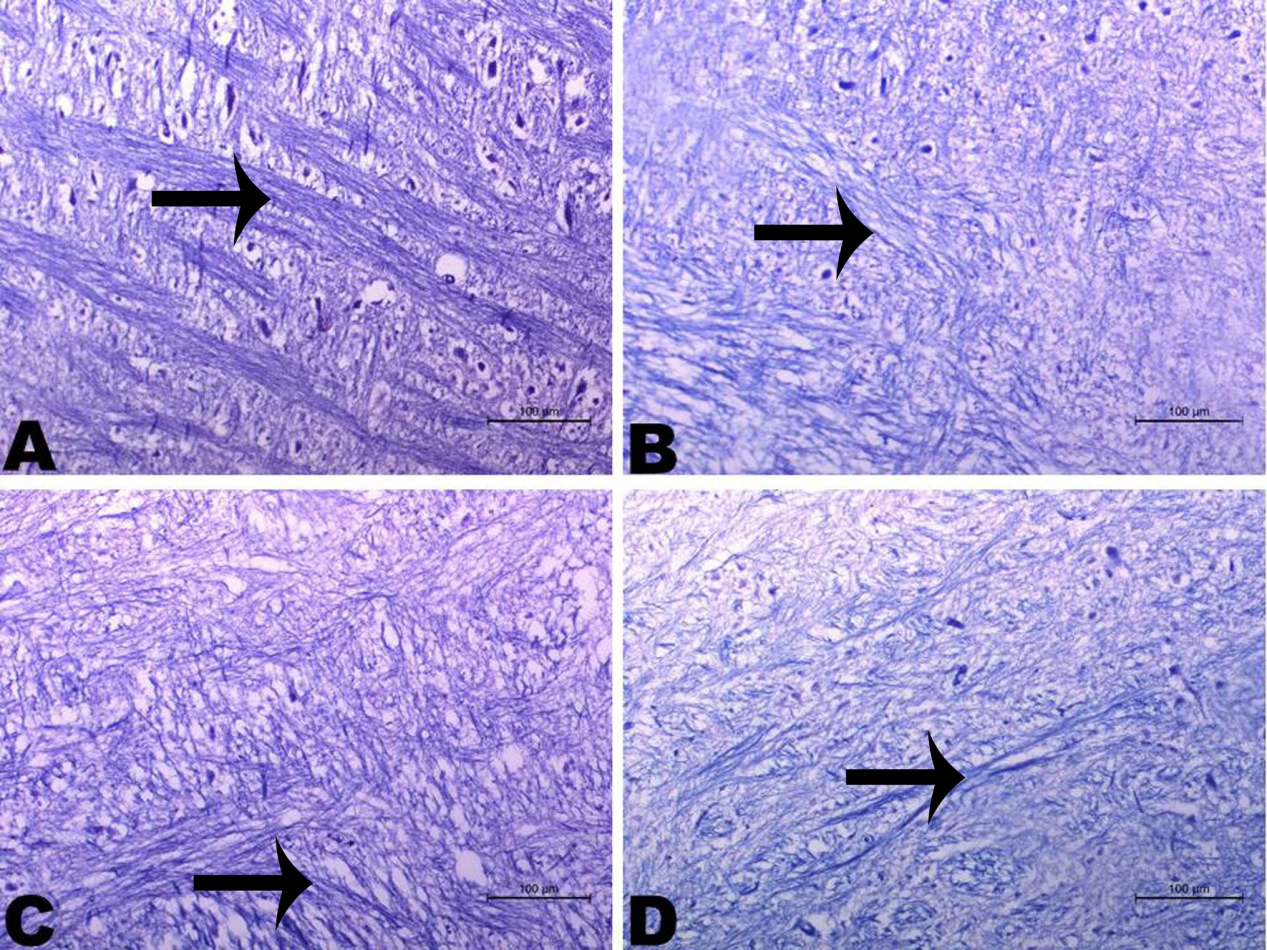
Histopathological changes of brain tissue in different studied groups. **A** Brain tissues of the normal control group showing long nerve fibers with continuous myelin sheath (arrow). **B** Brain tissues of AD-like untreated group showing the majority of nerve fibers appeared with irregular and ill myelinated (arrow). **C** Brain tissues of astaxanthin (AST) group showing nerve fibers with irregular myelin sheath (arrow). **D** Brain tissues of AST-LI formulation group with long nerve fibers with continuous myelin sheath (arrow). Luxol Fast Blue stain × 200

As demonstrated in Figs. 11B, 12B, and 13B, the brain tissues in the AD-like untreated group showed the hippocampal tissue with disrupted cell layers. The neurons and neuroglia cells suffered from degenerative changes and shrinkage. The brain’s vessels appeared highly congested with accumulation of acidophilic amyloid constituents in the hippocampal tissue and inside the neurons and neuroglia cells, as well as the majority of nerve fibers appeared irregular and ill myelinated. Treatment with AST-LI formulations significantly presented an improving effect by the return of hippocampal tissue to normalcy, as shown in Figs. 11, 12, and 13 (C and D) with more superior effects compared to free AST treatment.

## Discussion

The onset of AD neurodegeneration has been linked to aluminum intoxication. According to Sharma et al. [3] and Song [4], aluminum deposition in the central nervous system causes irreversible disruption of neuronal integrity and cognitive loss. In the present study, AlCl_3_ is essential for AD modeling because it can dramatically decrease the y-maze test’s working memory and the non-spatial memory in the NOR test in rats with the AD model when compared to control rats (Fig. 5). These results align with prior findings by Singh et al. [37] and Mohamed et al. [38]. Furthermore, Huang et al. [39] revealed a direct correlation between high levels of aluminum and cognitive impairment, especially in older people.

Cholinergic deficiency is a contributing factor to AD. In the current investigation, brain neurotransmitter changes were linked to the observed behavioral changes mediated by AlCl_3_. AD-like rats’ cerebral cortex and hippocampus had considerably higher AChE and MAO activities and significantly lower serotonin levels than control rats (Fig. 6). Al-induced neurotoxicity is caused by a number of interconnected mechanisms. Al^3+^ ions mimic Fe^3+^ and occupy iron-binding sites in transferrin, pass the BBB, and accumulate in the hippocampus and cortex as their size is comparable to that of Fe^3+^ ions [40]. This leads to alterations in cholinergic neurotransmission [41–43]. According to Zatta et al. [44], Al increases the enzymatic activity of AChE by modifying the peripheral locations and secondary structure of the enzyme, hence causing the brain’s ACh to be depleted, which is involved in learning and memory processes. Al also affects the serotonergic system, as demonstrated in the present study through the considerable decrease in serotonin levels and the triggering of the MAO enzyme (Fig. 6), thereby impairing memory and synaptic plasticity. MAO-A is a crucial mitochondrial flavoenzyme that aids in the breakdown of serotonin and norepinephrine. When monoamine neurotransmitters undergo oxidative deamination by MAO, aldehyde and hydrogen peroxide are produced, which are implicated in the development of amyloid plaques, inflammation, and the breakdown of neuronal membranes [45].

The findings of this investigation indicated that AST and AST-LI may function as inhibitors of AChE and MAO enzymes, as indicated by restoring the neurotransmitter balance via raising serotonin levels and decreasing AChE and MAO activities, thereby improving cognitive performance in behavioral (SAP, DI, and DR) scores compared to AD-like rats (Figs. 5 and 6). These results concurred with other research showing the cholinergic and serotonergic modulatory effects of AST. Rahman et al. [46] suggest AST’s role in neuromodulation. AST enhanced memory and decreased AChE activity in Aβ1-42-infused rats. AChE is also a dose-dependently inhibited by all-trans astaxanthin, according to Wang et al. [47]. Besides, AST raises serotonin levels in several brain areas, mimicking the actions of antidepressants [48]. These results point to the possible neuroprotective benefits of AST and AST-LI via cholinergic and serotonergic systems regulation and inhibition of AChE and MAO, in addition to raising monoamine levels.

Invasomes can improve the penetration of drugs potentially bypassing the blood–brain barrier and delivering therapeutic agents directly to the brain [15, 16]. This is particularly relevant for AD, where getting drugs to the brain is a major challenge. Their unique composition of phospholipids, ethanol, and penetration enhancers allows for increased membrane fluidity and elasticity, facilitating better penetration of biological barriers [15]. The higher capacity of AST-LI to improve spontaneous alternation behavior and recognition memory over those of free AST implies that lipid-based invasomal formulation may enhance brain permeability and brain targeting. According to our in vitro characterization, the optimum AST-LI was prepared using a formulation of phospholipid: ethanol: cineole as 300 mg: 0.3 ml: 0.1 ml for the production of stable vesicles with high entrapment efficiency in the presence of ethanol. In addition, the optimum zeta potential was a highly negative value indicating good physical stability and capacity to de-aggregate.

The pathophysiology of AD is significantly influenced by oxidative stress [49]. AlCl_3_ exposure exhibits strong pro-oxidant activity, disrupting the activities of antioxidant enzymes and compromising the redox balance[42, 50, 51]. Additionally, excessive ROS generation causes mitochondrial dysfunction, DNA, protein, and lipid oxidation, and other issues that contribute to the synthesis of more Aβ1-42, which in turn causes neuron death [49]. The current findings of low GSH levels and high MDA levels in the brains of AD-like rats (Fig. 7) indicate increased lipid peroxidation, which are consistent with those of Kazmi et al. [42]. These findings support the role of oxidative stress, neurotransmitter imbalances, and histopathological alteration in the hippocampal tissue, such as neuronal degeneration and amyloid accumulation in AD pathophysiology.

Alternatively, astaxanthin is a naturally occurring powerful antioxidant compared to other carotenoids and vitamin E [11]. Its unique chemical structure allows it to scavenge ROS and stop free radical chain reactions, thereby preventing oxidative DNA damage and lipid peroxidation. The strong antioxidant capability of AST and AST-LI treatment was demonstrated in this study by the markedly elevated GSH and lowered MDA levels leading to tissue improvement, which appeared in the histopathological findings (Fig. 7). These findings are consistent with those of Donoso et al. and Utomo et al. [11, 52] who reported that AST treatment may prevent dementia by improving the brain antioxidant status.

In addition to the cholinergic deficit, AD pathology involves the amyloidogenic cascade, marked by intracellular p-tau hyperphosphorylation and extracellular accumulation of misfolded Aβ protein as amyloid plaques. Such processes lead to the microtubule disruption and subsequent formation of NFTs within neurons [1, 5]. The disruption of various signaling pathways, including the SIRT-1/BDNF/miRNA-134 axis, plays a crucial role in the pathogenesis of AD. In the current investigation, AlCl_3_-induced neurotoxicity in AD-like rats led to increased levels of Aβ1-42, p-tau, GSK-3β, and GFAP along with decreased levels of SIRT-1, BDNF, and CX3CL1 proteins, causing neuronal degeneration and amyloid deposition in the nerve cells of the hippocampus, which was investigated by the histopathological findings. Additionally, miRNA-134, a crucial regulator of synaptic plasticity, was upregulated (Figs. 8 and 9).

Sirtuin 1 (SIRT-1), a nicotinamide adenine dinucleotide (NAD^+^)-dependent histone deacetylase, is essential for neuronal survival, cognitive function, and synaptic plasticity by controlling apoptosis, neurogenesis, autophagy, and oxidative stress [53]. SIRT-1 promotes chromatin opening for transcription by deacetylating histone proteins, in that way promoting neuronal survival and cognitive function [54]. SIRT-1 exerts neuroprotective effects through multiple mechanisms, including the reduction of oxidative stress and elevation of antioxidants like superoxide dismutase (SOD) and catalase via forkhead box O (FOXO) deacetylation [55, 56] and nuclear factor erythroid 2-related factor 2 (Nrf2) activation [57]. Lipid kinase phosphoinositide 3-kinase (PI3K) controls cell migration, differentiation, proliferation, and apoptosis through the activation of the downstream target protein kinase B (also known as PKB or AKT). SIRT-1 has been shown to increase neuronal survival and mitochondrial activity by activating the PI3K/Akt pathway, which in turn suppresses phosphorylated GSK-3β [58], decreasing tau phosphorylation and the production of Aβ peptides [59]. One important mechanism is that SIRT-1 directly upregulates BDNF expression by deacetylating the cAMP response element-binding protein (CREB) coactivator TORC1, triggering the CREB-TORC1 pathway, thereby enhancing synaptic plasticity and memory formation [60]. BDNF-induced activation/phosphorylation of CREB indirectly inhibits GSK-3β by activating AKT.

Glycogen synthase kinase 3 beta (GSK-3β) is a critical serine/threonine kinase mediator linking Tau pathology with Aβ buildup in AD. Elevated Aβ levels further enhance GSK-3β activity, exacerbating neurodegeneration [61]. Once activated, GSK-3β causes the tau protein to become hyperphosphorylated at serine residues, which destabilizes the tau protein and ultimately causes NFTs to develop [62]. GSK-3β can also enhance Aβ production by promoting the β-site amyloid precursor protein cleaving enzyme (BACE1) transcription, which is a β-secretase that sequentially cleaves amyloid precursor protein (APP) with the aid of γ-secretase to produce Aβ [1, 63]. Additionally, the PI3K/Akt pathway, essential for neuronal survival, is inhibited in AD, contributing to GSK-3β-increased tau phosphorylation and Aβ plaque accumulation as well as neuroinflammation [64, 65]. In the AlCl3-treated group, there was a significant increase in GSK-3β level, indicating its role in AD-related cognitive deficits. On the other hand, removing Aβ from the brain has a neuroprotective impact by downregulating GSK-3β and activating the PI3K-Akt axis. Treatments with AST and its nano-formulation (AST-LI) improve these disruptions, with AST-LI showing superior therapeutic effects (Figs. 8 and 9). AST as a SIRT-1 activator promotes neuronal survival and reduces neuroinflammation by activating PI3K/Akt [66] and blocking GSK-3β activity [46], which suppresses tau hyperphosphorylation and lowers NFTs formation [67]. Magadmi et al. [68] reported that AST successfully reversed scopolamine-induced reductions in Akt and phosphorylated Akt (p-Akt) in mice. Notably, AST lowers Aβ levels by inhibiting BACE1 transcription, reinforcing its role in mitigating AD pathology [69]. The current findings suggest that the neuroprotective actions of astaxanthin may be mediated through the SIRT-1/Akt/GSK-3β signaling.

Another mechanism of SIRT-1 is the control of miRNA-134 via a Yin Yang 1 (YY1)-containing repressor complex [70, 71]. Down-regulation of miRNA-134, which also targets SIRT-1 mRNA, results in overexpression of CREB, BDNF, Nrf2, and PI3K essential for synaptic plasticity, cognitive function, and neuroprotection [72]. Accordingly, SIRT-1-upregulated BDNF supports learning, memory, dendritic spine density, and neurite outgrowth while mitigating oxidative stress-induced brain aging. The results of this investigation are in line with previous studies reporting a reduction in BDNF expression after administering AlCl_3_ [41, 42].

The BBB-crossing lipid-soluble pigment AST promotes SIRT-1 expression [57], which in turn promotes BDNF upregulation and synaptic plasticity restoration in AD models [73]. Accordingly, Baby et al. [72] explained that the reduced expression of miRNA-134 can alleviate plasticity deficit in AD by increasing the expression of its downstream genes, containing CREB and BDNF. SIRT-1 directly works together with Yin Yang 1 (YY1) to suppress miRNA-134 expression and consequently enhances synaptic plasticity and memory through a posttranscriptional mechanism [71].

In the present study, it appears that downregulation of SIRT-1 and the elevation of miRNA-134 are responsible for the downregulation of BDNF mRNA and protein expressions in AD-like rat hippocampus neurons, which in turn impairs synaptic plasticity. Treatment with AST and AST-LI reversed these alterations, restoring BDNF levels (Fig. 9) and synaptic plasticity, further emphasizing the therapeutic and functional linking of SIRT-1/BDNF/miRNA-134/GSK-3β signaling in AD management.

Chronic activation of microglia and reactive astrogliosis are hallmarks of neurodegenerative disorders such as Alzheimer’s [50, 74, 75], leading to sustained release of pro-inflammatory cytokines and contributing to neuronal damage [75, 76]. The chemokine CX3CL1 (fractalkine), expressed in the central nervous system, plays a key role in regulating neuroinflammation by suppressing microglial activity through its receptor CX3CR1 [77], which is primarily present on microglia [78]. This CX3CL1 signaling pathway supports neuronal survival and synaptic plasticity by suppressing microglial activation and pro-inflammatory mediators such as IL-1β, IL-6, and TNF-α [79]. Down-regulation of CX3CL1 and BDNF and upregulation of GFAP, which is a marker of astrocytic activation, as reported in our study (Figs. 8 and 9), further indicate that neuroinflammation is related to learning and memory deficits of AlCl3-treated rats, supported by evidence that β-secretase (BACE1) contributes to fractalkine cleavage [80].

Importantly, treatment with AST and AST-LI significantly restored brain levels of CX3CL1 and GFAP compared to AD-like controls (Fig. 8), suggesting a possible mechanism for AST’s anti-inflammatory effect through fractalkine pathway activation [81, 82] and suppression of astrocytic activation [83, 84]. Such modulation may underlie the observed improvements in oxidative stress, histopathological markers, and cognitive outcomes, highlighting AST’s multifaceted neuroprotective role in AD, which are likely mediated through SIRT-1 activation.

The apoptotic cascade in AD is regulated by pro-apoptotic (BAX) and anti-apoptotic (BCL-2) proteins [85]. The ability of AlCl_3_ to induce oxidative stress, mitochondrial dysfunction, and cytoskeleton changes that lead to cell death or apoptosis is what causes the neurotoxicity in this study. This was indicated by upregulating BAX, BAX/BCL2 ratio, and caspase-3 and downregulating BCL-2 expressions (Fig. 10). On the other hand, the administration of AST and AST-LI to AD-like rats was linked to anti-apoptotic activity, as demonstrated by restoring BCL-2 expression and suppressing caspase-3 activation, BAX, and BAX/BCL2 levels. The ability of AST to suppress apoptosis has been attributed to SIRT-1 activation. These findings align with previous reports stating that SIRT-1 activators inhibit apoptosis through deacetylation of p53 and FOXO proteins [86]. Furthermore, its activation of the PI3K/Akt pathway modulates bad phosphorylation and downregulates cytochrome C release and caspase-3 [87]. BAX activation, caspases, and inflammatory factor expressions were all suppressed by AST treatment [88].

The histological data that were observed in this study matched the biochemical and behavioral findings. AlCl_3_-induced neurotoxicity, memory impairment, and apoptosis were all supported by the different histopathological changes (neuronal degeneration, shrinkage, disrupted cell layers, congested brain’s vessels, accumulation of acidophilic amyloid materials, and irregular-ill myelinated nerve fibers) observed in brain and hippocampal tissues of AD-like group (Figs. 11B, 12B, and 13B) as compared to controls (Figs. 11A, 12A, and 13A). Lesions in the brain and hippocampal regions resemble those described by Haider et al. [89] and Sumathi et al. [90]. Treatment with free AST partially protected the hippocampal tissues which is obvious in the mild degeneration, few cell layers, and the minimal amyloid deposition in the neurons and neuroglia of hippocampus (Figs. 11C, 12C, and 13C). The current findings demonstrated that the AD-like rats given AST-LI exhibited notable improvement at almost all levels of disease progress (Figs. 11D, 12D, and 13D). Similar results on the behavioral tests were linked to the reported AST’s improving effects on histopathological lesions. Wu et al. [73] demonstrated that AST considerably reduced the histological alterations in the aging rats’ hippocampus tissue.

The results highlight the neuroprotective potential of AST and AST-LI in modifying important AD pathways, with AST-LI showing improved effectiveness than free AST. This implies that AST-LI formulations may have a promising role in reducing AD-related cognitive impairments, possibly due to enhanced AST bioavailability and brain targeting offered by the invasomal delivery system.

## Limitations and Future Directions

This study demonstrated the neuroprotective effects of astaxanthin-loaded invasomes (AST-LI) in an Alzheimer’s disease rat model but has several limitations. Key pharmacokinetic parameters such as brain permeability, sustained release, and bioavailability were not assessed. Only male rats were used, limiting the generalizability due to potential sex-based differences. Long-term safety and toxicity were also not evaluated. Future studies should address these gaps by including pharmacokinetic analyses, both sexes, varied dosing, and chronic toxicity assessments to fully validate the therapeutic potential and translational applicability of AST-LI.

## Conclusion

Our results highlight the therapeutic potential of AST-LI as a SIRT-1 activator in reducing synaptic deficits and cognitive impairments linked to AD through the regulation of SIRT-1/BDNF/miRNA-134/GSK-3β axis that modulates the amyloidogenic, cholinergic, oxidative stress, neuroinflammatory, and apoptotic pathways. An additional mechanism of AST and AST-LI is the downregulation of GSK-3B-induced P-tau and Aβ plaques that underlie the observed improvements in neurotransmitter balance, behavioral scores, synaptic function, and cognitive loss linked to AD. Furthermore, AST-LI restored GFAP, CX3CL1, and apoptotic markers. AST-LI revealed superior neuroprotective benefits compared to free AST, possibly due to improved bioavailability, offering hope for effective management of AD-related cognitive impairments. Future research should further clarify the specific pharmacokinetic analyses, both sexes incorporation, and long-term effectiveness and therapeutic usefulness of AST-based formulations in AD therapy.

## Data Availability

The data used to support the findings of this study are available from the corresponding author upon request.

## Abbreviations

Al: Aluminum
AD: Alzheimer’s disease
Aβ: Amyloid β
SIRT-1: Sirtuin-1
AlCl3: Aluminum chloride
MDA: Malondialdehyde
CAT: Catalase
MAO: Monoamine oxidase
BDNF: Brain-derived neurotrophic factor
GFAP: Glial fibrillary acidic protein
GSK-3β: Glycogen synthase kinase 3 beta
BCL2: B cell leukemia lymphoma-2 protein
BAX: Bcl-2-associated protein x
GSH: Reduced glutathione
miRNA-134: Micro-ribonucleic acid-134
CX3CL1: Chemokine C-X3-C motif ligand 1
